# Caregiving Responsibilities and Mental Health Outcomes in Young Adult Carers during the COVID-19 Pandemic: A Longitudinal Study

**DOI:** 10.3390/ijerph192215149

**Published:** 2022-11-17

**Authors:** Giulia Landi, Kenneth I. Pakenham, Roberto Cattivelli, Silvana Grandi, Eliana Tossani

**Affiliations:** 1 Department of Psychology “Renzo Canestrari”, University of Bologna, 40127 Bologna, Italy; 2 Laboratory of Psychosomatics and Clinimetrics, Department of Psychology, University of Bologna, 47521 Cesena, Italy; 3 School of Psychology, The University of Queensland, Brisbane, QLD 4072, Australia

**Keywords:** young adult carers, emerging adulthood, parental illness, pandemic, caregiving responsibilities, mental health, wellbeing

## Abstract

This study investigated caregiving responsibilities and associated mental health outcomes in young adult carers during the COVID-19 pandemic and had three aims: (1) to investigate differences in caregiving responsibilities across two groups of young adult carers (parental illness context vs. ill non-parent family member context) relative to non-carers, (2) to identify COVID-19/lockdown correlates of caregiving responsibilities, and (3) to examine the longitudinal associations between caregiving responsibilities and mental health outcomes. Of the 1048 Italians aged 18–29 (*M*age = 24.48, *SD*age = 2.80; 74.33% female) who consented to complete online surveys at Time 1, 813 reported no ill family member (non-carers). Young adult carers included 162 with an ill parent and 73 with an ill non-parent family member. The study included 3 time points: 740 participants completed Time 2 assessment (*M*age = 24.35, *SD*age = 2.81; 76.76% female), while 279 completed Time 3 assessment (*M*age = 24.78, *SD*age = 2.72; 79.93% female). Key variables measured were 13 COVID-19/lockdown factors at Times 1 and 2, caregiving responsibilities at Time 2, and mental health outcomes at Time 3 (fear of COVID-19, anxiety, depression, wellbeing). Two COVID-19/lockdown factors were significantly correlated with higher caregiving responsibilities: insufficient home space, and greater time spent working and learning from home. As predicted, young adult carers reported higher caregiving responsibilities than non-carers, and this effect was greater in young adults caring for an ill parent compared to young adults caring for an ill non-parent family member. As expected, irrespective of family health status, caregiving responsibilities were longitudinally related to poorer mental health outcomes, operationalised as higher fear of COVID-19, anxiety, and depression, and lower wellbeing. Elevated young adult caregiving is an emerging significant public health issue that should be addressed through a multipronged approach that includes education about young adult carer needs for personnel across all relevant sectors and flexible care plans for ill family members that include a ’whole family’ biopsychosocial approach.

## 1. Introduction

Youth who take on responsibilities related to caring for a family member with disability, serious physical or mental illness, or alcohol/substance use problems, or who provide assistance to elderly relatives who have lost their autonomy due to age are broadly referred to as young carers [1,2]. Evidence indicates that, compared to their non-caring peers, these youth are at elevated risk for poorer mental health as well as other adverse psychosocial outcomes, such as poorer education, economic hardship, and worse health, especially when they care for an ill parent [3,4,5,6]. While many of these studies have examined children and adolescent carers up to 18 years, carers in the emerging adult developmental stage (age 18–25), referred to as ‘young adult carers’, have received remarkably less research attention [7,8]. The prevalence of young adult carers in Western societies ranges from 5.30–21.53% [9]. However, these prevalence figures are likely to be underestimates, as many youth do not identify as carers [2,10]. Young adult carers are expected to reach critically important milestones, including increasing autonomy, identity exploration, career development, and navigating intimate relationships [11], while also facing the challenges of caring for an ill family member. Two recent reviews indicated that young adult carers are expected to assume more caregiving responsibilities compared to child and adolescent carers, as they were considered by their families more reliable and able to deal with the strain and workload related to caregiving [8,9]. These reviews further demonstrated that in young adults, the caregiving role is associated with struggles around individual growth, planning for the future, maintaining a healthy lifestyle, and accessing education and employment opportunities, which negatively reverberate into adulthood.

According to Arnett [12], emerging adulthood has five defining features: identity exploration (search for meaning in ideologies, work, and relationships), instability (frequent changes in residences, jobs, and relationships), opportunities (increased options regarding career and life directions), self-focus (reduced obligations to family members and increased freedom to focus on self), and feeling in-between (the transitioning from adolescence to full adulthood). How these features intersect and fluctuate in prominence varies across time within and between young adults [13]. In addition, socio-demographic factors such as culture and socioeconomic status can influence how individuals manage these aspects of emerging adulthood and master associated challenges such as autonomy, identity formation, career progression, and intimate relationships. The workload, time, and interpersonal demands related to elevated caregiving during emerging adulthood can interfere with the smooth and timely navigation through this developmental period. Indeed, transactions with one’s living context, such as the presence of a seriously ill family member, can have a marked influence on one of the key tasks in emerging adulthood, identity formation [14]. 

Youth caregiving has been conceptualized as a continuum, ranging from undertaking small amounts of caregiving responsibilities—such as basic household chores—to increasingly higher amounts of caregiving duties at the expense of developmental needs [10,15,16]. Caregiving responsibilities refer to the psychological sense of duty or responsibility related to roles involved in contributing to family functioning [2,15]. An empirically tested model of youth caregiving posits caregiving responsibilities as a central element [17,18]. In addition, it constitutes a key factor in frameworks that account for how the illness of a family member impacts young carers [10,16,17,18]. Previous studies conducted with mixed samples that included child, adolescent, and young adult carers, revealed that higher caregiving responsibilities were associated with higher perceived chronic stress and, ultimately, poorer mental health [17,18].

In the context of the COVID-19 pandemic, caregiving responsibilities and activities are likely to be intensified due to the disruptions in healthcare systems and the social isolation caused by lockdown measures. COVID-19 related stressors, such as loneliness and uncertainties about the future, and the anxiety related to COVID-19 infection and transmission to the ill family member add to the already stressful circumstances of young carers [19]. We conducted a cross-sectional study during the second lockdown in Italy, which revealed that, compared to non-carers, young adult carers had poorer mental health [7], and this was most pronounced when the ill family member was a parent. Regarding the latter, prior studies of child, adolescent, and young adult carers have also found that, compared to young carers of non-parent ill family members, those who care for an ill parent report more mental health problems [20], as well as greater caregiving responsibilities [16]. We have previously argued that, given parents typically assume the primary caregiving role in relation to their children, a reversal of this role is likely to create more caregiving demands for youth caring for a parent than for those caring for a non-parent family member. This reversal of roles, whereby the child acts in the parenting role, is called parentification. Young adult carers have been found to report higher parentification than non-carer peers [21]. 

Our earlier young adult carer study mentioned above [7] further identified COVID-19 and lockdown factors that were significantly related to poorer mental health in young adult carers as follows: insufficient home space, currently in red zone, current COVID-19 infection, family member infected with COVID-19, family member death due to COVID-19, and reduced income [7]. However, this study did not examine whether these same COVID-19 and lockdown context factors are related to caregiving responsibilities in young adult carers. Hence, one aim of the present study is to investigate COVID-19 and lockdown correlates of caregiving responsibilities in young adult carers. To further explore prior caregiving research in young adult carer samples, we examined the two reviews on young adult carers [8,9], as well as more recently published manuscripts, and found ten quantitative studies that focused exclusively on young adult carers [21,22,23,24,25,26,27,28,29,30]. However, none of these studies had examined caregiving responsibilities using a validated and targeted self-report scale, and none employed a longitudinal methodology. Hence, the purpose of this study is to investigate the longitudinal associations between young adult carer caregiving responsibilities, using a validated scale, and mental health outcomes in the context of the COVID-19 pandemic.

### The Present Study

The objective of the present study is to increase our understanding of caregiving responsibilities and their associations with mental health outcomes in young adult carers during the COVID-19 pandemic. To this purpose, the aims of the present study are threefold:

1. To investigate differences in caregiving responsibilities in the context of the COVID-19 pandemic across three groups of young adults: carers in a parental illness context, those caring for an ill non-parent family member, and non-carers. Informed by prior research, we predicted that young adult carers would report higher caregiving responsibilities than non-carer peers and that young adult carers of an ill parent would report higher caregiving responsibilities than those caring for an ill non-parent family member.
2. To identify socio-demographic and COVID-19 context and lockdown contextual elements that are associated with higher caregiving responsibilities in young adult carers relative to non-carers.
3. To examine the longitudinal association between caregiving responsibilities and mental health outcomes (operationalized as fear of COVID-19, anxiety, depression, and wellbeing) after controlling for the effects of family health status (parental vs. other ill family member) and relevant socio-demographic and COVID-19 context variables. Based on the findings from previous research, we predicted that higher caregiving responsibilities would predict subsequent poorer mental health outcomes.

## 2. Materials and Methods

### 2.1. Participants and Recruitment Procedure

This study has three assessment points, three months apart. The first was conducted between 19 January 2021 and 2 February 2021, during the second Italian mandatory lockdown, in which most Italian Regions had been classified in the previous two months into one of two risk categories: ‘red’ zone (i.e., some restrictions in the first 2020 strict national lockdown) or ‘orange’ zone (i.e., people could go to work, but most non-essential industries were closed, and people could not leave their province). The second assessment was conducted between 19 April and 3 May 2021, a period in which all restrictions were in place and gradually relaxed toward the end (i.e., on 26 April ‘yellow’ zones where “open-air business activities” could reopen). The third assessment occurred between 19 July and 2 August 2021, a period in which all restrictions were lifted, and all Italian Regions were in the low restriction ‘white’ zone. For the purposes of this study, we refer to these three assessment points as Time 1, Time 2, and Time 3, respectively. 

**Figure 1:**
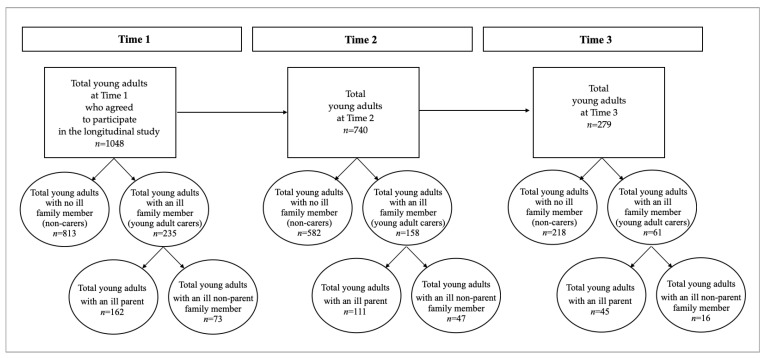
Flow Chart Displaying Participant Enrolment at Each Assessment Point.

As with our prior cross-sectional study of young adult carers during COVID-19 (mentioned above) [7], the present study is based on data collected from a larger longitudinal project investigating protective psychological resources during the COVID-19 pandemic. A total of 3626 participants from the community were enrolled through social media and a snowballing approach and took part in the first online survey conducted during the second Italian mandatory lockdown. After removal of careless responders—i.e., those who answered incorrectly to one or both of the following attention check items: “To demonstrate your attention, select the answer agree a little”, and “To demonstrate your attention, select the answer never”—the sample was composed of 3341 participants. Of these, 1823 were young adults aged 18–29 years old, and of these, 1048 consented to take part in the longitudinal project and constituted the final total young adult sample for this study. At each assessment, participants completed an online questionnaire through the Qualtrics software, which took 15–20 min to complete. The inclusion criteria for this longitudinal study were being aged 18–29 years, resident in Italy, and consenting to participate in the longitudinal study. It was not possible to calculate an accurate response rate because recruitment was primarily conducted through social networks. This study was approved by the Ethics Committee of the University of Bologna. A flow chart displaying participant enrolment at each assessment point is presented in Figure 1.

Although most research on young adult carers has examined the 18–25 years age range, for the present study we focused on the 18–29 age range. There is widely published data showing that in high-income countries emerging adulthood typically extends up to age 29, with parallel trends towards longer time in education and later entry into marriage and parenthood [11]. This revised extended developmental period for emerging adulthood is now widely used (e.g., see review, [31]). Finally, most studies on young adult carers have recruited from higher education student populations or from organisations that support formally identified young carers and young adult carers. In the present study, we recruited young adults from the general community with no requirement for participants to self-identify as a carer. Regarding the latter, given the evidence that young people in caregiving roles do not always identify as carers [2,32], investigating only those who self-identify as carers may exclude young people who have substantial care responsibilities but do not identify as a carer.

### 2.2. Measures

#### 2.2.1. Family Health Status

Participants reported at Time 1 whether any person they were living with had a serious physical or mental health condition. If ‘yes’, they indicated who was ill (either a parent or another ill family member). Two variables were subsequently created: parental illness (0 = *no* and 1 = *yes*) and other ill family member (0 = *no* and 1 = *yes*). At Time 1, 77.58% of young adults (*n* = 813) reported no family member with a serious health condition (labelled the non-carer group), and 22.42% (*n* = 235) reported an ill family member in their household. Of the latter, 15.46% (*n* = 162) reported living with a parent with a serious health condition (labelled the parental illness [PI] group), and 6.97% (*n* = 73) reported living with a non-parent family member with a serious health condition (labelled the other ill family member [OIFM] group). Other ill family members were comprised of grandparents (*n* = 72, 6.87%), and siblings (*n* = 50, 4.77%), with the remainder being some other ill family member (i.e., uncles/aunts or cousins). The number of participants in each of the sub-groups (PI, OIFM, non-carers) at each assessment point are summarized in Figure 1.

#### 2.2.2. Socio-Demographics

At Time 1, participants reported their gender, age, education, marital status, occupation, socio-economic status (SES), and nationality. They further indicated if they had children and if they had a physical health condition. Finally, if they reported living with an ill family member, participants reported their illness type (i.e., physical or mental). 

#### 2.2.3. COVID-19 and Lockdown Context Variables

The following variables were assessed to obtain information on participants’ experiences related to COVID-19 and lockdown at Time 1: (1) participants’ perceptions of the adequacy of home space during the mandatory restrictions was evaluated with the following item rated on a 5-point scale (0 = *not at all* to 4 = *very much*): “Is the size of your home insufficient to guarantee your personal space, despite the mandatory lockdown, such as number of rooms in relation to the people you live with?”; (2) Number of people in the household during the lockdown; (3) To assess potential reductions in family income due to the COVID-19 pandemic, participants indicated whether they (a) lost work, (b) were receiving a redundancy payment, or (c) if their income substantially decreased in other ways due to COVID-19 restrictions (endorsement of one or more of the three items was scored as 1 = *yes*, no endorsement of all three items was scored as 0 = *no*); (4) Participants rated the amount of time they spent working from home (‘smart working’) or in distance learning from the beginning of the second lockdown on a 5-point scale (0 *never in smart working or distance learning* to 4 *always in smart working or distance learning*); (5) participants reported whether they worked in a health-care setting; (6) participants’ perceptions of structural change to their social network was evaluated with the following item rated on a 5-point scale (0 = *no change* to 4 = *extremely reduced*): “How has the size of your social network (e.g., friends, family, relatives, or social events with colleagues) changed compared to life before COVID-19?”. 

Additionally, the following variables related to direct experiences with lockdowns, COVID-19, and vaccination were assessed at Time 1 and Time 2 due to potential changes in these variables across the two assessment points: (7) Participants estimated the percentage (0% to 100%) of time they spent in a ‘red’ zone at both Times 1 and 2 (the mean time spent in a ‘red’ zone was calculated by averaging the scores across these two items). They also reported at both Times 1 and 2 whether they were (8) infected by COVID-19, if they (9) required hospitalization, as well as (10) whether family members were infected, (11) hospitalized, or (12) deceased due to COVID-19; (13) Finally, participants reported whether they had received the COVID-19 vaccine. Endorsement of each of these categorical variables related to direct experiences with lockdowns, COVID-19, and vaccination assessed at Time 1 or Time 2, or both time points (i.e., COVID-19 infected, COVID-19 hospitalized, family member infected, family member hospitalized, family member death, and COVID-19 vaccine), was scored as 1 = *yes*, and no endorsement at the two time points was scored as 0 = *no*.

#### 2.2.4. Caregiving Responsibilities

Caregiving responsibilities were assessed at Time 2 with the Caregiving Responsibilities subscale of the Italian version [32] of the Young Carer of Parents Inventory-Revised [2,15]. It consists of 7 items (e.g., “My parent(s) relies on me to help them with household chores”, “I have to look after my other family members”, and “My parent(s) expects me to help care for them”) rated on a 5-point scale (0 *strongly disagree* to 4 *strongly agree*). Scores were averaged with higher scores reflecting greater caregiving responsibilities. The subscale demonstrated good internal reliability and validity in the derivation [2,15] and Italian validation studies [32]. The Caregiving Responsibilities scale has also been validated in a tripartite model of youth caregiving, which posits caregiving responsibilities as one of three central constructs (the other two being caregiving experiences and caregiving tasks) that constitute youth caregiving. Importantly, the Caregiving Responsibilities scale has been applied across the youth caregiving continuum and has been validated in varying caregiving contexts, including the presence and absence of a serious ill family member [10]. The caregiving responsibilities subscale has been widely used as an independent predictor in prior young carers research [10,16,17,18]. The observed McDonald’s omega was 0.80. 

#### 2.2.5. Mental Health Outcomes

##### Fear of COVID-19

Fear of COVID-19 was measured at Time 3 with the validated Italian version [33] of the Fear of COVID-19 Scale (FCS) [34]. The FCS is composed of 7 items measuring emotional, cognitive, physiological, and behavioural manifestations of fear related to COVID-19. Each item (e.g., “It makes me uncomfortable to think about coronavirus-19”) is rated on a 5-point Likert scale (1 *strongly disagree* to 5 *strongly agree*). Item scores are summed, with higher scores reflecting higher fear of COVID-19 (range 7–35). Previous studies have reported good psychometric properties [34]. The observed McDonald’s omega was 0.87. 

##### Anxiety

Anxiety was assessed at Time 3 with the validated Italian version [35] of the General Anxiety Disorder Scale (GAD-7) [36]. The GAD-7 questionnaire measures anxiety symptoms over the past two weeks (i.e., “Not being able to stop or control worrying” or “Feeling afraid as if something awful might happen”). Items are rated on a 4-point Likert scale (0 *not at all* to 3 *nearly every day*). Item scores are summed, with higher scores reflecting higher anxiety. The instrument has been shown to be psychometrically sound [37]. The observed McDonald’s omega was 0.90.

##### Depression

The validated Italian version [38] of the Patient Health Questionnaire (PHQ-9) [39] was used to measure depressive symptomatology at Time 3. Items are rated referring to the past two weeks (i.e., “Feeling down, depressed, or hopeless” or “Feeling bad about yourself—or that you are a failure or have let yourself or your family down”) on a 4-point Likert scale (0 *not at all* to 3 *nearly every day*). All item scores are summed, with higher scores indicating higher depression. The measure has demonstrated sound psychometric properties [40]. The observed McDonald’s omega was 0.88.

##### Wellbeing

The validated Italian version [41] of the Mental Health Continuum Short Form (MHC-SF) [42] was used to assess wellbeing at Time 3. This 14-item scale evaluates emotional (i.e., “How often did you feel happy?”; 3 items), psychological (i.e., “How often did you feel good at managing the responsibilities of your daily life?”; 6 items), and social (i.e., “How often did you feel that you belonged to a community?”; 5 items) wellbeing. Items are rated on a 6-point Likert scale (0 *never* to 6 *every day*) with the last month as the timeframe. Items were summed with higher scores indicating higher wellbeing. The measure has demonstrated sound psychometric properties [43]. The observed McDonald’s omega was 0.91.

### 2.3. Data Analysis Approach

Descriptive statistics and reliability tests of study variables were conducted in IBM SPSS 24, while regressions and correlations were run in M*plus* 8.4 with the robust maximum likelihood estimator (MLR) [44]. The overall percentage of missing data on each study scale across all three assessments was 14.12%. Little’s missing completely at random test [45] on the variables of interest yielded a normed χ^2^ (χ^2^/df) of 1.429. This index, which can be used to correct for sensitivity of the χ^2^ for large samples [46], is low and suggests that data are missing completely at random. Based on the results of these preliminary analyses, the complete baseline sample (N = 1048) was retained, and the full information maximum likelihood estimator available in *Mplus* was used to handle missing data.

To investigate the first aim, one regression was conducted to examine differences in Time 2 caregiving responsibilities across the three groups (PI, OIFM, and non-carer). The endogenous variable was Time 2 caregiving responsibilities while the exogenous variables were the two family health status dummy variables (i.e., PI and OIFM). Because the non-carer group was represented as a score of 0 on both dummy variables, the regression intercept equalled the mean score on the outcome variable for the non-carer group. The regression coefficients for the PI and OIFM dummies provide a test of the mean differences between the two young adult carer groups and non-carers, while controlling for the presence of OIFM or PI, respectively. Regression analysis was conducted controlling for any socio-demographic variables that significantly differed among the three groups (PI, OIFM, non-carers). 

To examine the second aim, we conducted correlations between both Time 1 socio-demographic and Times 1 and 2 COVID-19 context variables and Time 2 caregiving responsibilities in the PI, OIFM, total young adult carer, and non-carer groups.

To explore the third aim, four linear regressions were conducted to examine the longitudinal association between caregiving responsibilities and mental health outcomes. For each model, the endogenous variable was one of the four mental health outcomes at Time 3 (i.e., fear of COVID-19, anxiety, depression, and wellbeing), while the exogenous variable was Time 2 caregiving responsibilities together with the confounders (i.e., the two family health status dummy variables (PI, OIFM) and socio-demographic and COVID-19 context variables that were significantly correlated with Time 2 caregiving responsibilities), as well as any socio-demographic variables that significantly differed among the three groups (PI, OIFM, non-carers). Local effect size in multiple regressions was calculated with Cohen’s ƒ^2^ [47], with effect sizes of 0.02, 0.15, and 0.35 considered as small, medium, and large effects, respectively [48]. 

## 3. Results

### 3.1. Preliminary Data Analyses

#### 3.1.1. Sample Characteristics

Table 1 presents the socio-demographics and COVID-19 and lockdown context variables as well as descriptive data on caregiving responsibilities for the PI, OIFM, non-carer groups, and total sample. The total longitudinal sample consisted of 1048 young adults at Time 1 (74.33% female; Time 1 *M*age = 24.48, *SD*age = 2.80). Half of the participants (52.67%) reported having a bachelor’s degree, 39.89% secondary school, and 2.86% primary school levels, while 4.58% completed a postgraduate course. A small proportion of the sample (11.30%) reported being married or living with a partner, while the remainder reported being single (88.70%). Over half of the participants were currently studying (59.64%), 33.87% were currently working, and 9.82% reported not being in education, employment, or training. Regarding socio-economic status, most of the sample reported a mean income between EUR 15,001–36,000 (43.03%), 29.87% a mean income between EUR 36,000–70.000, while 17.18% and 9.92% reported a mean income in the lower (<EUR 15,000) and upper (>EUR 70.000) levels, respectively. Almost all participants (97.61%) were of Italian nationality.

We further investigated whether the three groups (PI, OIFM, non-carers) differed on socio-demographic variables. The only significant differences were in marital status, χ^2^(2, 1048) *=* 15.59, *p* = 0.000, and currently working, χ^2^(2, 1048) *=* 11.03, *p* = 0.004. Specifically, compared to the non-carer group, the PI group had fewer participants who reported they were married or living with a partner (PI 3.09%, non-carers 13.28%) and currently working (PI 23.46%, non-carers 36.41%). We controlled for these variables in multivariate analyses.

#### 3.1.2. Attrition

Figure 1 presents the number of participants who completed each of the three assessment points. Of the total Time 1 sample, 76.72% of participants (*n* = 804) responded to two assessments and 20.52% (*n* = 215) to all three data collection points. To examine attrition, we conducted a series of ANOVAs and Chi-squared tests, which compared respondents who participated in at least two assessments with those who only completed the Time 1 assessment. There were no significant differences between the two groups in family health status (PI vs. OIFM vs. non-carer), χ^2^[2, 1048] *=* 3.42, *p* = 0.18. Furthermore, the two groups did not differ on most socio-demographics and COVID-19 and lockdown context variables. However, according to the Chi-squared test values, there were significant differences between the two groups in gender, employment, and family member hospitalized, although the standardized residuals were low (<2), indicating that the differences were negligible. The only exception was a marginal overrepresentation of participants reporting higher amounts of time in smart working or distance learning among those who participated in at least two assessments (χ^2^[1, 1046] *=* 11.28, *p* = 0.001, Cohen’s *d* = 0.13). Nevertheless, according to Cohen’s guidelines, the effect size for this significant difference is small (i.e., Cohen’s < 0.20 is considered small; [48]). 

### 3.2. Differences in Caregiving Responsibilities among PI, OIFM and Non-Carer Groups

Regarding the first study aim, we conducted a linear regression to test the prediction that young adult carers would report higher caregiving responsibilities than non-carer peers and that young adult carers of an ill parent (PI group) would report higher caregiving responsibilities than those caring for an ill non-parent family member (OIFM group). The non-carer group was represented by a score of 0 on both PI and OIFM dummy coded variables; therefore, the standardized coefficient for PI indicates a test of the mean differences between PI and non-carers (controlling for the presence of OIFM), while the standardized coefficient for OIFM provided a test of the mean differences between OIFM and non-carers (controlling for the presence of PI). Analyses were conducted by controlling for the two socio-demographic variables that significantly differ across the three groups (i.e., marital status and currently working). Results indicated that, compared to non-carers, both the PI group and the OIFM group reported higher caregiving responsibilities (β = 0.125, *p* < 0.001, and β = 0.098, *p* < 0.05, respectively). The effect of the PI group was significantly stronger than the effect of the OIFM group with a medium effect for PI (Cohen’s ƒ^2^ = 0.016) compared to a small effect for OIFM (Cohen’s ƒ^2^ = 0.010). Results were subjected to posteriori power analyses for linear multiple regressions. Results of these analyses revealed that, given a sample size of 1048, an α of 0.05, and the effect sizes obtained in caregiving responsibilities for the PI and the OIFM groups (i.e., 0.016 and 0.010, respectively), the resulting power (1-β) calculations were 0.94 for the PI group and 0.78 for the OIFM group, respectively.

### 3.3. Correlations between Time 1 Socio-Demographics and Times 1 and 2 COVID-19 Context Variables and Time 2 Caregiving Responsibilities

Regarding the second study aim, we conducted correlations to identify socio-demographic and COVID-19 and lockdown contextual elements that are associated with higher caregiving responsibilities in young adult carers relative to non-carers. Correlations between Time 1 socio-demographics and Times 1 and 2 COVID-19 context variables and Time 2 caregiving responsibilities in the PI, OIFM, total young carer, and non-carer groups are reported in Table 2. Almost all correlations in the young adult carer groups were non-significant. Only insufficient home dimension was significantly related to Time 2 caregiving responsibilities in the total young adult carer group (*r* = 0.18, *p* < 0.05). In addition, in the OIFM group, two significant correlations emerged with Time 2 caregiving responsibilities. In particular, age was positively associated with Time 2 caregiving responsibilities (*r* = 0.41, *p* < 0.05), while working and studying from home was negatively correlated to Time 2 caregiving responsibilities (*r* = −0.29, *p* < 0.05). As for the non-carer group, low socio-economic status was positively correlated to Time 2 caregiving responsibilities (*r* = 0.10, *p* < 0.05).

Significant correlations were subjected to posteriori power analyses. In the OIFM group, given a sample size of 87, an α of 0.05, and the obtained significant correlation coefficients of 0.41, and 0.29, respectively, the resulting power (1-β) calculations were 0.98 for the relationship between age and caregiving responsibilities and 0.87 for the relationship between working or studying from home and caregiving responsibilities. In the total young adult carer group, given a sample size of 235, an α of 0.05, and the obtained significant correlation coefficient of 0.18, the resulting power (1-β) calculation was 0.88 for the relationship between insufficient home space and caregiving responsibilities. Finally, in the non-carers group, given a sample size of 813, an α of 0.05, and the obtained significant correlation coefficient of 0.10, the resulting power (1-β) calculation was 0.88 in the relationship between low socio-economic status and caregiving responsibilities.

### 3.4. Longitudinal Associations between Caregiving Responsibilities and Mental Health Outcomes

Regarding the third study aim, we tested the hypothesis that higher caregiving responsibilities would predict subsequent poorer mental health outcomes by conducting four linear regressions, which examined the association between Time 2 caregiving responsibilities and Time 3 mental health outcomes. The regressions controlled for the two family health status dummy coded variables [PI, OIFM] and the socio-demographic and COVID-19 context variables that were significantly correlated with Time 2 caregiving responsibilities (i.e., age, insufficient home dimension, working and studying from home) as well the two socio-demographic variables that significantly differed across the three groups (i.e., marital status and currently working). Standardized beta coefficients and Cohen’s ƒ^2^ for each mental health outcome are presented in Table 3. Findings showed that, irrespective of family health status (PI, OIFM, non-carer), higher Time 2 caregiving responsibilities were associated with poorer mental health outcomes at Time 3: fear of COVID-19, *β* = 0.153, *p* < 0.05; anxiety, *β* = 0.194, *p* < 0.01; depression, *β* = 0.158, *p* < 0.01; wellbeing, *β* = −0.214, *p* < 0.01. The effect size of caregiving responsibilities was medium for fear of COVID-19 and depression (Cohen’s ƒ^2^ = 0.024, and ƒ^2^ = 0.026, respectively) and large for anxiety (Cohen’s ƒ^2^ = 0.041) and wellbeing (Cohen’s ƒ^2^ = 0.048). Results were subjected to a posteriori power analyses for linear multiple regressions. Results of these analyses revealed that, given a sample size of 1048, an α of 0.05, and the effect sizes obtained for caregiving responsibilities of 0.02 for fear of COVID-19, 0.04 for anxiety, 0.03 for depression, and 0.05 for wellbeing, the resulting power (1-β) calculations were 0.96, 0.99, 0.97, and 0.99, for fear of COVID-19, anxiety, depression, and wellbeing, respectively.

## 4. Discussion

This study aimed to: (1) investigate differences in caregiving responsibilities across two groups of young adult carers (those in a parental illness context and those caring for an ill non-parent family member) relative to non-carers, (2) identify COVID-19 and lockdown correlates of caregiving responsibilities, and (3) examine the longitudinal associations between caregiving responsibilities and mental health outcomes during the COVID-19 pandemic. As expected, compared to non-carers, young adult carers reported higher caregiving responsibilities, and this effect was greater in young adults caring for an ill parent compared to young adults caring for an ill non-parent family member. Regarding the second aim, two COVID-19 and lockdown contextual elements emerged as significant correlates of higher caregiving responsibilities in young adult carers: insufficient home space and greater time spent in smart working or distance learning. Finally, as predicted, caregiving responsibilities were longitudinally related to poorer mental health outcomes, irrespective of family health status.

The results indicating that young adult carers reported higher caregiving responsibilities compared to non-carers, and that this effect was greater in young adults caring for an ill parent compared to young adults caring for an ill non-parent family member, are consistent with findings from previous studies that included mixed carer samples of child, adolescent, and young adult carers [2,10,15,16,32]. However, the present study is the first to highlight these relationships in a sample composed of only young adult carers in a pandemic context. When a family member has a serious illness, families often meet illness demands by redistributing roles among family members, which often includes youth taking on more family caregiving activities [2]. However, compared to their child and adolescent siblings, young adult carers tend to assume more caregiving responsibilities as they are considered ‘older’ and more autonomous by their families and more able to cope with the stressors associated with caregiving [8,9]. Young adult carers are therefore likely to take on more of the adult caregiving roles and responsibilities than other youth in the family, particularly if the ill family member is a parent.

Two COVID-19 context and lockdown contextual elements were identified in this study as significant correlates of higher caregiving responsibilities in young adult carers: perceptions of insufficient home space in the total young adult carer group and greater time spent in smart working or in distance learning in the group of young adult carers caring for an ill non-parent family member. Insufficient home space during the COVID-19 lockdowns was highlighted in our previous study [7] as a correlate of higher fear of COVID-19 in young adult carers of an ill non-parent family member. We previously speculated that, compared to non-carers, young adult carers are likely to experience more fear of COVID-19 due to the added concerns about the health vulnerabilities of the ill family member and, therefore, spend more time at home to avoid infection, which may, in turn, result in a sense of restlessness due to feeling confined to a relatively small living space. Spending more time at home working or undertaking distance learning where the living space is perceived as insufficient is also likely to result in less opportunities for the young adult to escape caregiving tasks. This may account for the associations between both insufficient home space and working and learning from home and higher caregiving responsibilities. 

Finally, as expected, irrespective of family health status, caregiving responsibilities were longitudinally related to poorer outcomes across the wellbeing and distress (fear of COVID-19, anxiety, and depression) dimensions of mental health. These findings are consistent with those of prior studies that have used mixed young carer samples (child, adolescent, and young adult carers), and various study deigns, including cross-sectional and longitudinal predictive methodologies and model testing investigations exploring the impacts of an ill family member on youth [10,16,17,18,20,32]. A consistent finding from these studies is the link between higher levels of caregiving responsibilities and poorer mental health. The results of the present study expand on this body of research by demonstrating this link in an exclusively young adult carer sample, using a longitudinal study design, in the context of the COVID-19 pandemic. 

The COVID-19 and lockdown related findings from the present study show that more time working or learning at home and insufficient home space are related to higher caregiving responsibilities, which in turn is related to more COVID-19-related fear. These results converge with the COVID-19 and lockdown findings from our prior study, which showed that young adult carers reported poorer mental health across COVID-19-related and general mental health outcomes than non-carers, and that a notable minority of young adult carers reported clinically significant levels of distress as well as elevated levels of pandemic-related family violence, fear of COVID-19, risky health behaviours, and loneliness [7]. In addition, six COVID-19 and lockdown factors were related to poorer mental health: insufficient home space, currently in red zone, current COVID-19 infection, family member infected with COVID-19, family member death due to COVID-19, and reduced income. The findings from both studies suggest that during a pandemic, young adult carers are particularly vulnerable to the development or exacerbation of mental health problems and that caregiving responsibilities and a range of pandemic contextual factors function as potential mental health risk factors. 

There are several potential pathways by which caregiving responsibilities may adversely affect the mental health of young adult carers. The wide range of potentially demanding caregiving tasks that young adult carers undertake can impose restrictions on the amount of time and type of activities they engage in within education, employment, social, and recreational settings [2]. Restricted engagement in activities in these areas can reduce skill development and increase isolation, which, in turn, are associated with vulnerability to mental health problems. Caregiving pressures can conflict with the demands of establishing a career and elicit decision-making conflicts regarding future life planning relative to their ongoing caregiving predicament [8,9]. Young adult carers may worry about the ill family member and feel guilty when they pursue their own interests [2]. The redistribution of caregiving tasks in the family can strain family relations and reduce the amount of family support [18]. These challenges and stressors can interfere with the timely and smooth accomplishment of emerging adulthood milestones (e.g., autonomy, identity formation, career development, and intimate relationships) and lead to mental health problems. As mentioned in the introduction, the healthcare system disruptions and social isolation caused by the COVID-19 pandemic intensify caregiving responsibilities [19]. In addition, COVID-19 related stressors and anxiety are likely to exacerbate the abovementioned mental health challenges.

In taking a cultural lens to the results of this study, it is important to note how youth and family culture in Italy may shape the experience of caregiving during emerging adulthood. For example, Italian youth tend to prolong academic study, delay entry into the labour market, live at home for a protracted period, and move out of their family homes predominantly for marriage and parenthood [49]. Family ties also tend to be very strong in Italy and other Mediterranean countries [50,51]. It is possible that these youth and family cultural factors may intensify the caregiving experience of young adults who care for a seriously ill family member. To the extent that these Italian youth and family cultural factors are not prominent in other cultures, findings from the present study may not generalize to some countries. However, as noted above, the pattern of findings from this study related to youth caregiving responsibilities and mental health outcomes are consistent with the results of similar studies conducted across many different countries.

Elevated young adult caregiving is a significant public health issue given the global rise in the number of young adults caring for an ill or disabled family member [51], the often-hidden nature of youth caregiving, the vulnerability of emerging adults navigating critical development milestones, and the association between youth caregiving and greater risks for mental health problems. Results from the present study show that, during the COVID-19 pandemic, caregiving responsibilities remain elevated in young adult carers relative to non-carer peers and confirm the link between higher levels of caregiving responsibilities and poorer mental health. Hence, young adult caregiving should be targeted by preventive mental health services, particularly during a pandemic. Young adult carers engage with a wide range of sectors including education, vocational training, employment, and health. Personnel across these areas should be educated about young adult carer needs and procedures for identifying and responding to them and how to make necessary supports available within their organization [16]. In addition, flexible alternative care and supports for ill family members, particularly parents, are necessary to lessen the caregiving load placed on young adult carers. These should be delivered across a number of areas depending on priority needs (e.g., physical, medical, psychological, practical, and financial). Many healthcare professionals do not routinely collect information regarding the family status and composition of medical patients and, instead, hold a narrow focus on the patient’s physical symptoms and disability. A broader biopsychosocial approach is required, which includes the family context. As such, a ‘whole family approach’ to the provision of coordinated assessments and support services for the person with care needs and their family, including the young carer, is likely to ease youth caregiving strain. The ‘whole family approach’ is an orientation toward healthcare that includes the family unit where appropriate and has been used to inform government policy in the provision of welfare and health services in many developed counties. It has been recommended as a guiding framework for assisting young carers in many parts of the world, including UK [52], Europe [53], Canada [54], and USA [55]. It has also been used to inform the development of specific empirically based supportive interventions for ill parents and their children [56]. Based on the findings from the present study and those from our related prior study, the provision of support services for young adult carers, such as those mentioned above and in our earlier study, are critical for promoting the mental health of young carers, particularly during a pandemic.

Limitations of the present study include the following: First, our online convenience sampling limits the generalizability of findings to the general population of young adult carers. Specifically, the present sample had an overrepresentation of people with the following socio-demographic characteristics: higher education, middle socio-economic status, and being female. Nevertheless, the proportion of young adults who were identified as carers is consistent with that reported by previous research in the young adult carer field [9]. Second, the reliance on self-report measures increases the risk of common method variance. Third, attrition at Time 3 was relatively high (79.48%), although drop-out at Time 2 was markedly lower (23.28%). Fourth, we examined a limited range of COVID-19 and lockdown risk factors, and, therefore, we may have omitted risk factors that are particularly potent for some young adult carers. Fifth, caregiving responsibilities were only measured at one time point. Sixth, the illness severity of the ill family member was not assessed and is likely to be related to young adult carer caregiving responsibilities. Seventh, due to the assessment of mental health outcomes at one timepoint, the causal relations between caregiving responsibilities and mental health outcomes remain ambiguous. Finally, according to the tripartite model of youth caregiving [10], caregiving responsibilities is only one of three caregiving constructs (the others being caregiving experiences, and caregiving tasks). Future studies should examine the impact of all three components of youth caregiving in order to more fully explore the mechanisms by which caregiving impacts the mental health of young adult carers. The strengths of this study include (1) the longitudinal design, (2) the assessment of both the distress and wellbeing dimensions of mental health, (3) the use of a validated measure of caregiving responsibilities, (4) a sample of young adult carers drawn from the community rather than higher education student populations, as has occurred in most prior young adult carer studies, (5) the assessment of family health status (PI vs. OIFM), which has been neglected in prior research, (6) the investigation of young adult carer mental health relative to a range of COVID-19 pandemic factors, and (7) the use of a young adult age range that better reflects the duration of emerging adulthood in contemporary society within developed countries. 

## 5. Conclusions

The objective of the present study was to investigate caregiving responsibilities and their longitudinal association with mental health outcomes in young adult carers in the context of the COVID-19 pandemic. To this end, the study yielded three important findings. First, as predicted, young adult carers reported higher caregiving responsibilities than non-carers, and this effect was greater in young adults caring for an ill parent compared to those caring for an ill non-parent family member. This finding highlights the importance of the family member status of the ill person, a factor often ignored in prior young adult carer research. Results converge with other research findings suggesting that youth caring for a parent are more at risk than those caring for an ill non-parent family member. Second, of the 13 COVID-19 and lockdown factors investigated, only insufficient home space and greater time spent in smart working or distance learning were associated with higher caregiving responsibilities in young adult carers. The common element across both factors is the family living environment, which suggests a potential target for support services. Finally, as predicted, higher levels of caregiving responsibilities longitudinally predicted poorer mental health outcomes, irrespective of family health status. Consistent with this result are findings from studies that have tested models of youth caregiving and theories of how family member illness impacts youth adjustment. Such studies have identified caregiving responsibilities as a key explanatory mechanism in shaping young carer mental health. Elevated young adult caregiving is an emerging significant public health issue that should be addressed through a multipronged approach that includes education about young adult carer needs for personnel across all sectors frequently accessed by young adults and flexible care plans for ill family members that include a ‘whole family’ biopsychosocial approach.

## Figures and Tables

**Table 1:** Descriptive Data on Time 1 Socio-demographics, Times 1 and 2 COVID-19 Context Variables, and T2 Caregiving Responsibilities in the Parental Illness (PI), Other Ill Family Member (OIFM), and Non-carer Groups.

	PI (*n* = 162)		OIFM (*n* = 73)		Non-Carers (*n* = 813)		Total Sample (N = 1048)	
Variable	%	*M* (*SD*)	%	*M* (*SD*)	%	*M* (*SD*)	%	*M* (*SD*)
*Socio-demographics*								
Gender: female	75.31		76.71		73.92		74.33	
Age years		24.10 (2.65)		24.29 (2.66)		24.57 (2.84)		24.48 (2.80)
Education								
Primary school	2.47		1.37		3.08		2.86	
Secondary school	42.59		42.47		39.11		39.89	
Bachelor’s degree	50.62		49.32		53.38		52.67	
Postgraduate course	4.32		6.85		4.43		4.58	
Single	96.27		92.78		86.97		88.70	
Married or living with a partner	3.09		6.85		13.28		11.26	
Currently working	23.46		28.77		36.41		33.87	
Currently studying	67.90		61.64		57.81		59.64	
Not in education, employment, or training	9.88		12.32		9.59		9.82	
Socio-economic status								
<EUR 15,000	25.31		15.07		15.74		17.18	
EUR 15,001–36,000	43.83		38.36		43.30		43.03	
EUR 36,000–70,000	21.60		38.36		30.75		29.87	
>EUR 70,000	9.26		8.22		10.21		9.92	
Italian nationality	98.15		98.63		97.42		97.61	
Presence of a physical health condition	11.11		16.44		8.86		9.73	
Ill family member illness type—physical	66.05		56.16		-		14.03	
Ill family member illness type—mental	46.29		45.21		-		11.07	
*COVID-19 and lockdown context variables*								
Insufficient home dimension ^a^		1.39 (1.01)		1.51 (1.16)		1.34 (1.01)		1.36 (1.02)
Number of people in the household		3.44 (1.08)		3.92 (1.19)		3.11 (1.18)		3.22 (1.18)
Reduced family income	11.11		9.60		8.00		8.59	
Working or studying from home ^a^		2.68 (1.79)		2.37 (1.82)		2.32 (1.84)		2.38 (1.84)
Work in a health-care setting	11.11		10.96		11.56		11.92	
Structural change to social network ^b^		1.97 (1.27)		2.01 (1.38)		2.01 (1.27)		2.01 (1.27)
Percentage of time spent in red zone		34.23 (22.39)		32.74 (24.25)		33.02 (23.69)		33.97 (24.02)
COVID-19 infected (T1 + T2)	19.75		9.59		15.50		16.27	
COVID-19 hospitalized (T1 + T2)	0.00		0.00		0.00		0.00	
Family member infected (T1 + T2)	38.22		23.29		33.03		33.13	
Family member hospitalized (T1 + T2)	10.49		4.11		8.61		8.59	
Family member death (T1 + T2)	7.41		5.48		10.95		8.49	
COVID-19 vaccine (T1 + T2)	17.35		12.82		19.60		18.84	
*T2 caregiving responsibilities*		2.16 (0.71)		2.11 (0.77)		1.89 (0.72)		1.95 (0.73)

Note. ^a^ Variables coded on a 5-point Likert scale 0 = *not at all or never* to 4 = *very much or always*. ^b^ Variable coded on a 5-point Likert scale 0 = *no change* to 4 = *extremely reduced*. T1 = Time 1, T2 = Time 2.

**Table 2:** Correlations between Time 1 Socio-demographics and Times 1 and 2 COVID-19 Context Variables and Time 2 Caregiving Responsibilities in the Parental Illness (PI), Other Ill Family Member (OIFM), Total Young Adult Carer (YAC), and Non-carer Groups.

	Time 2 Caregiving Responsibilities			
	PI (*n* = 111)	OIFM (*n* = 47)	YACs (*n* = 158)	Non-Carers (*n* = 582)
*Socio-demographics*				
Gender (1 = female)	−0.037	0.047	−0.011	−0.072
Age	0.019	0.411 *	0.098	−0.064
Low education (1 = secondary school or below)	−0.001	0.122	0.085	0.020
Currently single ^¥^	−0.074	−0.143	−0.065	0.005
Currently working ^¥^	0.081	0.117	−0.117	0.009
Currently studying ^¥^	−0.390	0.112	−0.283	0.007
Not in education, employment, or training ^¥^	−0.151	0.113	0.121	0.060
Low socio-economic status (1 = < EUR 15,000)	0.017	−0.123	0.021	0.099 *
Nationality (1 = Italian)	−0.098	0.102	0.091	−0.017
Presence of a physical health condition ^¥^	−0.038	−0.092	−0.080	0.035
Family illness type—physical ^¥^	0.051	0.064	0.042	-
Family illness type—mental ^¥^	−0.042	−0.037	−0.042	-
*COVID−19 Context Variables*				
Insufficient home dimension	0.126	0.112	0.183 *	−0.039
Number of people in the household	−0.006	−0.011	0.022	0.061
Reduced family income ^¥^	0.039	0.152	0.128	0.081
Working or studying from home	0.018	−0.289 *	0.083	0.028
Work in a health-care setting ^¥^	0.013	0.162	0.094	0.010
Structural change to social network	0.184	−0.081	0.067	0.025
Percentage of time spent in red zone (T1 + T2)	−0.044	−0.055	−0.088	−0.050
COVID−19 infected (T1 + T2) ^¥^	−0.096	0.012	−0.096	0.050
Family member infected (T1 + T2) ^¥^	0.111	0.018	0.073	−0.084
Family member hospitalized (T1 + T2) ^¥^	−0.001	0.114	0.051	−0.019
Family member death (T1 + T2) ^¥^	−0.063	0.045	−0.031	−0.029
COVID-19 Vaccine (T1 + T2) ^¥^	0.126	−0.149	−0.028	−0.012

Note. * *p* < 0.05. ^¥^ Spearman’s correlations for categorical variables dummy coded as 1 = *yes*, 0 = *no*. T1 = Time 1, T2 = Time 2.

**Table 3:** Linear Regressions of Time 2 Caregiving Responsibilities Predicting Time 3 Mental Health Outcomes.

	Time 3 Fear of COVID-19	Time 3 Anxiety	Time 3 Depression	Time 3 Wellbeing
	*β* (ƒ^2^)	*β* (ƒ^2^)	*β* (ƒ^2^)	*β* (ƒ^2^)
Time 2 Caregiving responsibilities	0.153 * (0.024)	0.194 ** (0.039)	0.158 ** (0.026)	−0.214 ** (0.048)
*Family health status*				
PI ^†^	−0.020 (0.000)	0.026 (0.001)	0.088 (0.008)	−0.129 (0.017)
OIFM ^†^	−0.002 (0.000)	−0.053 (0.003)	−0.038 (0.001)	−0.014 (0.000)
*Other confounders*				
Age	0.009 (0.000)	−0.050 (0.003)	−0.124 (0.016)	0.065 (0.004)
Marital status	0.078 (0.006)	0.024 (0.001)	0.095 (0.009)	−0.133 (0.018)
Currently working	−0.134 (0.018)	−0.078 (0.006)	−0.072 (0.005)	0.247 ** (0.065)
Insufficient home dimension	−0.028 (0.001)	0.071 (0.005)	0.111 (0.012)	0.029 (0.001)
Working or studying from home	−0.073 (0.005)	0.103 (0.011)	0.086 (0.007)	−0.001 (0.000)

Note. * *p* < 0.05, ** *p* < 0.01. PI = parental illness, OIFM = other ill family member, *β =* standardized beta coefficient, ƒ^2^ = Cohen’s ƒ^2^. ^†^ The non-carer group was represented by a score of 0 on both PI and OIFM dummy variables, the standardized coefficient for PI indicates a test of the mean differences between PI and non-carers (controlling for the presence of OFMI), while the standardized coefficient for OIFM provided a test of the mean differences between OIFM and non-carers (controlling for the presence of PI).

## Data Availability

The dataset analysed during the current study is available from the corresponding author upon reasonable request.
